# Factors that Affect Pancreatic Islet Cell Autophagy in Adult Rats: Evaluation of a Calorie-Restricted Diet and a High-Fat Diet

**DOI:** 10.1371/journal.pone.0151104

**Published:** 2016-03-10

**Authors:** Qianqian Sun, Shuangshuang Nie, Lingxiao Wang, Fan Yang, Zhangming Meng, Hengyi Xiao, Bing Xiang, Xiujun Li, Xianghui Fu, Shuang Wang

**Affiliations:** 1 The Center of Gerontology and Geriatrics, West China Hospital, Sichuan University, Chengdu, Sichuan province, China; 2 Laboratory of Aging Research, West China Hospital, West China Medical School, Sichuan University, Chengdu, Sichuan province, China; 3 The Department of Hematology, West China Hospital, Sichuan University, Chengdu, Sichuan province, China; 4 The Department of Endocrinology and Metabolism, West China Hospital, Sichuan University, Chengdu, Sichuan province, China; 5 State Key Laboratory of Biotherapy and Cancer Center, West China Hospital, Sichuan University, and Collaborative Innovation Center for Biotherapy, Chengdu, Sichuan province, China; Hosptial Infantil Universitario Niño Jesús, CIBEROBN, SPAIN

## Abstract

Aging may be a risk factor for type 2 diabetes in the elderly. Dietary intervention can affect glucose tolerance in adults, which may be due to body composition and islet cell autophagy. The aim of this study was to determine the effects of various dietary interventions on islet cell autophagy. Pancreatic tissue and blood samples were collected from Sprague Dawley rats (14–16 months old, n = 15 for each group) that received a normal diet (ND), a high-fat diet (HFD), or a calorie-restricted diet (CRD). The body weight (BW), visceral fat, serum lipid levels, fasting serum glucose, insulin levels, and β/α cell area were determined in 14-16-(0-w), 16-18-(8-w), and 18-20(16-w)-month-old rats. Pancreatic islet autophagy (LC3B and LAMP2), AP (Acid Phosphatase) and apoptosis (apoptosis index, AI (TUNEL assay) and cleaved caspase-3) were detected using immunohistochemistry, ELISA and western blot. At 16 weeks, the expressions of LC3B, LAMP2 and AP markedly increased in both the HFD (P<0.01) and CRD (P<0.05) groups; however, an increase in the AI (P<0.05), cleaved caspase-3 and Beclin1 expression and a decrease in the expressions of BCL2 and BCLXL (P<0.05) were observed in only the HFD group. FFA, triglyceride levels, HOMA-IR, insulin levels and glucagon levels were significantly increased in the HFD group but decreased in the CRD group at 16 weeks (P<0.05). The degree of islet cell autophagy was potentially regulated by the levels of FFA and islet cell insulin and glucagon, which may have been due to the effects of Beclin1/BCL2.

## Introduction

Glucose metabolism reduces with age, resulting in impaired glucose tolerance and a high prevalence of type 2 diabetes (T2DM) in the elderly population[1]. Age-related increases in visceral adiposity and decreases in insulin sensitivity (IS) may promote the degeneration of islet cell function, which leads to a diabetic condition[2, 3]. Autophagy is crucial for the function and survival of pancreatic β cells by maintaining a balance between the synthesis, degradation, and subsequent recycling of cellular products[4–6], whereas autophagy may induce islet cell death through a direct (autophagic cell death) or indirect pathway (apoptosis)[7]. Dietary intervention can affect glucose tolerance via autophagy[8]. For example, a high-fat diet has been proven to increase insulin resistance and thereby promote the prevalence of T2DM[9, 10], and HFD intervention in young mice (8 weeks old) was shown to increase autophagic flux in pancreatic β cells both in and ex vivo[11]. A calorie-restricted diet (CRD) is considered the most robust non-genetic autophagic promoter and lifespan-extending method in many species[12]. Although cell autophagy has been implicated in physiological aging[13] and cellular stress responses[11], the relationships between the pancreatic islets and autophagy in addition to islet cell physiological aging and diabetes in adults have not been extensively explored. Furthermore, from a whole-body perspective, little is known regarding the relative effects of islet cell autophagy to the dietary-related changes in the body and β/α cells during aging. Therefore, our study evaluated the expression of autophagic proteins and changes in body composition, insulin and glucagon in islet cells after dietary intake intervention, including CRD and HFD, from mid to late adulthood in rats.

## Materials and Methods

### Animals

Forty-five healthy male Sprague Dawley (SD) rats (n = 15 per group) aged 14–16 months (weighing 500–550 g) were purchased from the animal center of Sichuan University. The rats were randomly grouped and received a normal diet (ND, 3.2 kcal/kg), a HFD (5.86 kcal/kg) or a CRD (containing 50% of the calorie content of the ND; Table 1) and were sacrificed at 0, 8, and 16 weeks, respectively.

**Table 1:** Composition and calorie content of the diet in the experimental groups.

Content	ND (3.2 kal/kg)/CRD (1.6 kal/kg) feeds		HFD feeds	
	Weight Ratio (wt/wt%)	Energy Ratio(%)	Weight Ratio (wt/wt%)	Energy Ratio(%)
Carbohydrate	53.77	66.5	22.68	15.48
Fat	3.67	10.21	43.27	66.43
Protein	18.83	23.29	26.51	18.08
Other	23.73		7.54	
Total	100	100	100	100

The animals were fasted for 12 h prior to sample collection. After measuring the body weight (BW), the animals were anesthetized with an intraperitoneal injection of 3% sodium pentobarbital (30 mg/kg). The blood was drawn from the heart and was stored at -20°C. The pancreas was also quickly collected, fixed and embedded in paraffin (for immunohistochemistry) or cut into pieces and ground in liquid nitrogen (for western blot). The visceral adipose tissues were collected and weighed. The body fat ratio was calculated using the following formula:

bodyfatratio=(weightofvisceralfat(g)⁄BW(g))×100%.

Our experimental protocol was approved by the Animal Research Protection Committee of the West China Hospital of Sichuan University.

### Biochemical analysis of blood glucose, plasma insulin, lipids and FFA levels and estimation of insulin resistance status

Fasting plasma glucose (FPG) was measured using the ACCU-CHEK active blood glucose meter (Roche, Basel, Switzerland), fasting insulin (Fins) was measured using a rat insulin radioimmunoassay kit (ELISA, Beijing North Institution Of Biological Technology, Beijing, China), triglycerides were measured using the GPO-POD assay (Sichuan Michael Technology Limited Liability Company, Sichuan, China), cholesterol levels were evaluated using an enzyme-coupled colorimetric assay (Sichuan Michael Technology Limited Liability Company, Sichuan, China), and FFA levels were measured using a colorimetric assay (Beijing Jiuqiang, Beijing, China).

The insulin sensitivity index (ISI), homeostatic model assessment of insulin resistance (HOMA-IR), and homeostatic model assessment of β cell function (HOMA-β) were calculated using the following formulae:

ISI=1⁄(FPG×Fins)

HOMA−IR=(FPG×Fins)⁄22.5

HOMA−β=20×Fins⁄(FPG−3.5)

### ELISA

The pancreatic tissues were collected and ground in liquid nitrogen with extraction lysis buffer (without phosphatase inhibitors). The protein concentration was determined using the BCA Kit, and the final concentration was 1 μg/ul. Acid phosphatase (AP) activity was quantified using Acid Phosphatase Assay ELISA Kit (Beyotime, Beijing, China). The pooled samples were measured in 3 repeat ELISA experiments.

### Immunohistochemistry

Dissected rat pancreases were rinsed in ice-cold 10 mM PBS and fixed in 4% paraformaldehyde. Immunohistochemical staining was performed in two steps. The sections were deparaffinized and rehydrated, and antigens were retrieved by autoclaving the slides in 10 mM citric acid buffer. The following antibodies were used: anti-insulin (1:200, Bioss, Beijing, China), anti-glucagon (1:200, Bioss, Beijing, China), anti-LC3B (1:200, Beyotime, Shanghai, China), anti-LAMP2 (1:200, Bioss, Beijing, China), anti-Beclin 1 (1:200, Bioss, Beijing, China), anti-BCL2 (1:200, Bioss, Beijing, China), anti-BCLXL (1:200, Abcam, USA) and anti-cleaved caspase-3 (1:200, Proteintech, Wuhan, China). After washing the slides twice with PBS (5 min/wash), they were incubated with biotinylated goat-anti-rabbit IgG (BioVision, Mountain View, CA, USA) secondary antibodies at 37°C for 40 min, followed by incubation with HRP-conjugated streptavidin (BioVision, Mountain View, CA, USA). The islets were visualized using an Olympus IX71 microscope, and the mean optical density (MOD) was analyzed using the Image-Pro Plus 5.0 software (Media Cybernetics, Rockville, MD, USA).

The area of the α/β cell in each islet was calculated from the glucagon- and insulin-positive cells, where

thearea=thenumberofpointscounted×thepointarea.

Two slides were selected from each animal, and five fields (400x) in each slide were randomly selected for calculating the MOD.

### Western blot analysis

The pancreatic tissue was collected and ground in liquid nitrogen. The samples were harvested into universal protein extraction lysis buffer (Cat. No. PP1801, Bioteke, Beijing, PR China) containing a protease inhibitor cocktail (Cat. No. 04693116001, Roche, Basel, Switzerland). After the protein concentration was determined using the BCA method (Bioteke, Beijing, China), 20–50 μg of total cell protein was separated via SDS-PAGE, transferred to Polyvinylidenefluoride (PVDF) membranes (Cat. No. IPVH00010, Millipore, Massachusetts, USA), and incubated with the appropriate primary antibodies and horseradish peroxidase-conjugated secondary antibodies. Specific proteins were visualized using an enhanced chemiluminescence (ECL) western blot detection system (Millipore). The optical densities of the bands were measured using Image Pro plus 6.0 (IPP 6.0, Media Cybernetics, Silver Springs, MD, USA) software. The relative expression levels were calculated as the ratio of the optical density of the target protein to GAPDH.

### TUNEL assay

The free 3’-OH groups in broken cellular DNA resulting from apoptotic degradation were detected using the TUNEL assay (terminal deoxynucleotidyl transferase-mediated dUTP nick-end labeling), which was performed according to the manufacturer’s instructions. Pancreatic tissue sections (4 μm) were mounted on a slide, dewaxed and rehydrated in graded alcohol solutions. Immunohistochemical detection and quantification of terminal transferase dUTP nick end labeling (TUNEL) were performed using an in situ cell death detection kit (TUNEL-POD; Roche, Basel, Switzerland) following the manufacturer’s instructions. Apoptotic cells (brown staining) were visualized using an Olympus microscope under 400x magnification. The apoptotic index (AI) was expressed as the means ± S.D. for the percentage of positive cells and was calculated using the formula:

AI=(apoptosis−positivecells⁄totalcells)×100%.

Two slides were selected from each animal, and five fields (400x) in each slide were randomly selected to quantify the positive cell number per 1,000 cells.

### Statistical analysis

The biochemical parameters are presented as the means ± S.D.. Statistical analyses were performed using one-way ANOVA with a Bonferroni correction. When a homogeneity of variance test could not be performed, we used transformations. All data analyses were performed using the SPSS 17.0 software. P-values<0.05 were considered statistically significant.

## Results

### Effects of CRD and HFD on islet cell autophagy (LC3B, LAMP2 and AP expression) in adult rats

**Fig 1:**
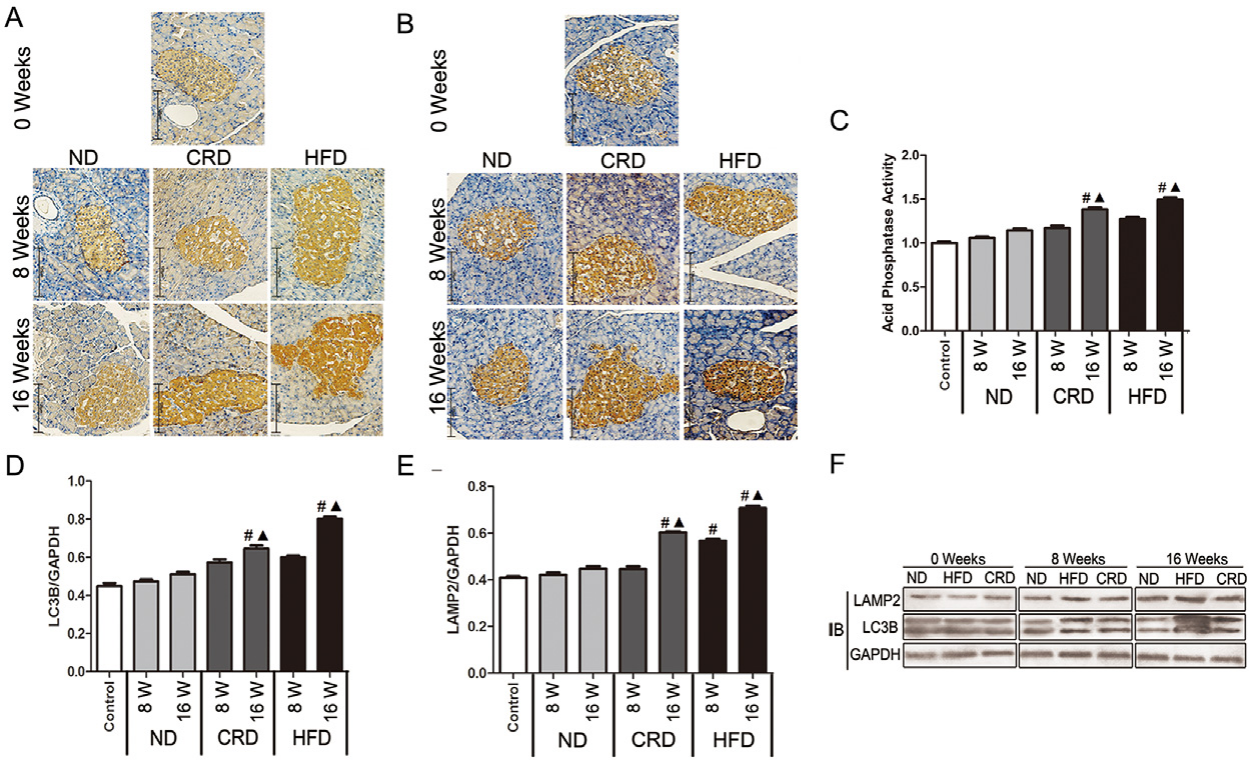
Effect of dietary intervention on islet cell LC3B, LAMP2 and AP expression in adult SD rats (n = 5 for each group). Immunohistochemical staining of LC3B (A), LAMP2 (B) (brown staining) in the ND, HFD and CRD groups at 0, 8 and 16 weeks (bars represent 125 μm), respectively. ELISA analysis of AP activity (C) in the pancreatic islets of adult SD rats fed a ND, HFD, or CRD. Expression of LC3B and LAMP2 were evaluated via Western blot (F) in the ND, HFD and CRD groups at 0, 8 and 16 weeks. Western blot analysis of LC3B (D) and LAMP2 (E). #: vs. 0 weeks, ▲: CRD/HFD vs. ND, ★: CRD vs. HFD. P<0.05. 0 weeks (14 months old), 8 weeks (16 months old), and 16 weeks (18 months old).

Pancreatic islet cell autophagy was determined by analyzing the levels of the autophagy-specific proteins (LC3B and LAMP2) using both immunohistochemistry and western blot analyses, and AP was determined via ELISA assay. As shown in Fig 1, compared with the ND-fed rats, a statistically significant upregulation of autophagic markers (LC3B and LAMP2) in both the HFD (P<0.01) and CRD (P<0.05) groups was observed. After 16 weeks of intervention, a statistically significant upregulation of AP in both the HFD (P<0.05) and CRD (P<0.05) groups was observed. There were no statistically significant differences between the HFD and CRD groups; however, the HFD group had a higher expression than the CRD group in the immunohistochemistry, western blot analyses and ELISA analyses.

### Effects of CRD and HFD on islet cell apoptosis (AI and c-caspase-3 expression) in adult rats

**Fig 2:**
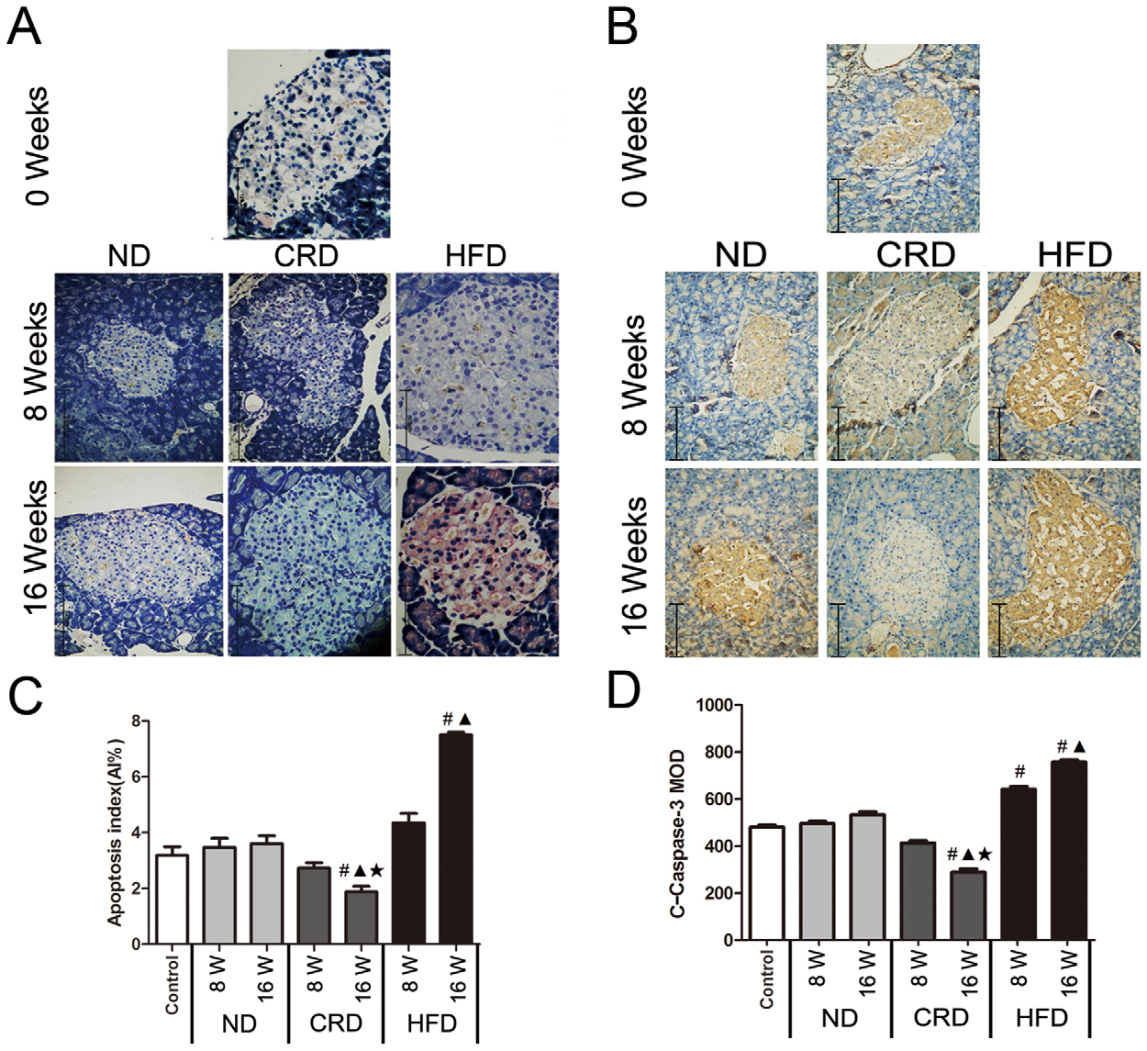
Effects of dietary intervention on islet cell apoptosis (AI and c-caspase-3) in adult SD rats (n = 5 for each group). TUNEL staining (A) and c-caspase-3(B) in the ND, HFD and CRD groups at 0, 8 and 16 weeks (bars represent 125 μm), respectively. Apoptosis index (C) and MOD analysis of c-caspase-3 (D) in the pancreatic islets of adult SD rats fed a ND, HFD, or CRD. #: vs. 0 weeks, **▲**: CRD/HFD vs. ND, ★CRD vs. HFD. P<0.05. 0 weeks (14 months old), 8 weeks (16 months old), and 16 weeks (18 months old).

Apoptosis was evaluated using the TUNEL assay (positive apoptotic cells stain a brown color) and analyzing the expression levels of the c-caspase-3 using immunohistochemistry analyses. As shown in Fig 2, After 16 weeks of intervention, a significant increase in AI (P<0.05) and c-caspase-3 (P<0.05) in the HFD-fed rats and a reduction of AI (P<0.05) and c-caspase-3 (P<0.05) in the CRD-fed rats were observed compared with the ND group.

### Effects of the CRD and HFD on Beclin1, BCL2 and BCLXL expression in adult rat islet cells

**Fig 3:**
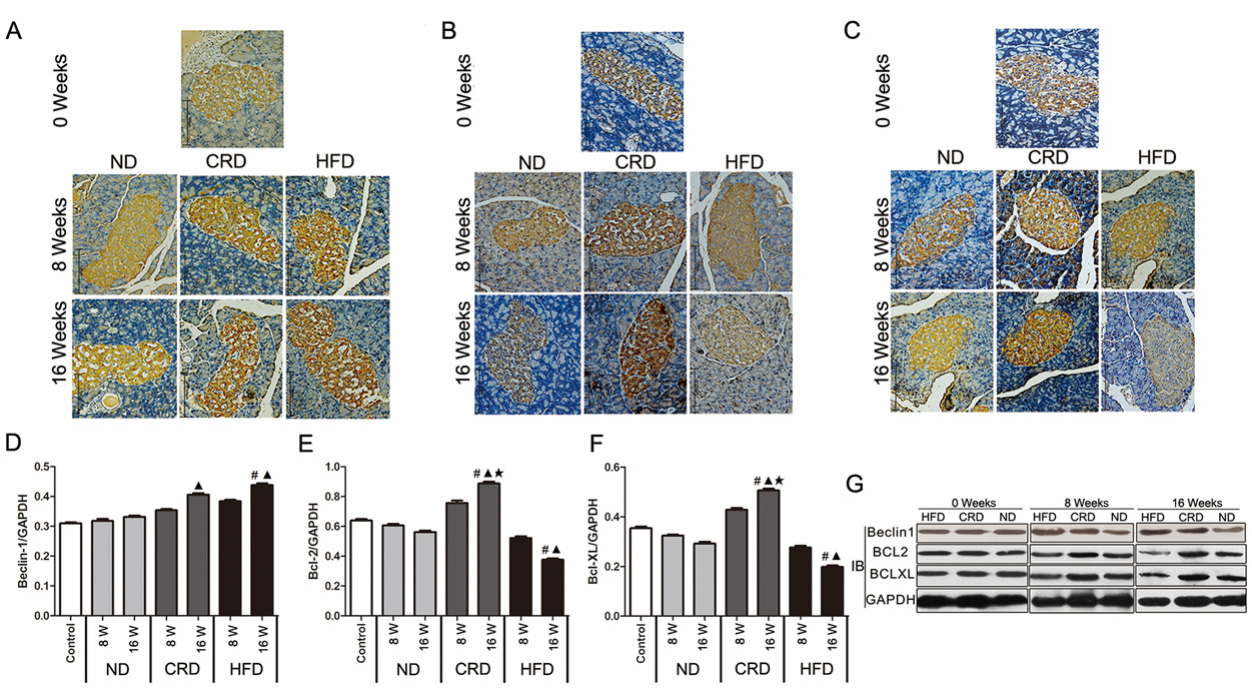
Effect of dietary intervention on the expressions of Beclin1, BCL2, and BCLXL (brown staining) in the islet cells of adult SD rats. Immunohistochemical staining of Beclin1 (A), BCL2 (B) and BCLXL (C) in the ND, HFD and CRD groups at 0, 8 and 16 weeks (bars represent 125 μm). Expression of Beclin1 (D), BCL2 (E), and BCLXL (F) evaluated via Western blot (G) in the ND, HFD and CRD groups at 0, 8 and 16 weeks. #: vs. 0 weeks, **▲**: CRD/HFD vs. ND, ★: CRD vs. HFD. P<0.05. 0 weeks (14 months old), 8 weeks (16 months old), and 16 weeks (18 months old).

The expression levels of Beclin1, BCL2 and BCLXL were assessed using both immunohistochemical staining and western blot. As shown in Fig 3, after 16 weeks of intervention, we found a statistically significant upregulation of Beclin1 in both the HFD (P<0.01) and CRD (P<0.05) rats compared with the ND-fed rats; however, no statistically significant differences between the HFD and CRD groups were observed. There was an observable decrease in the BCL2 and BCLXL expression (P<0.05) in the rats fed the HFD, whereas BCL2 and BCLXL were significantly increased in the rats fed the CRD.

### Effects of CRD and HFD on body composition and lipid levels in SD adult rats

**Fig 4:**
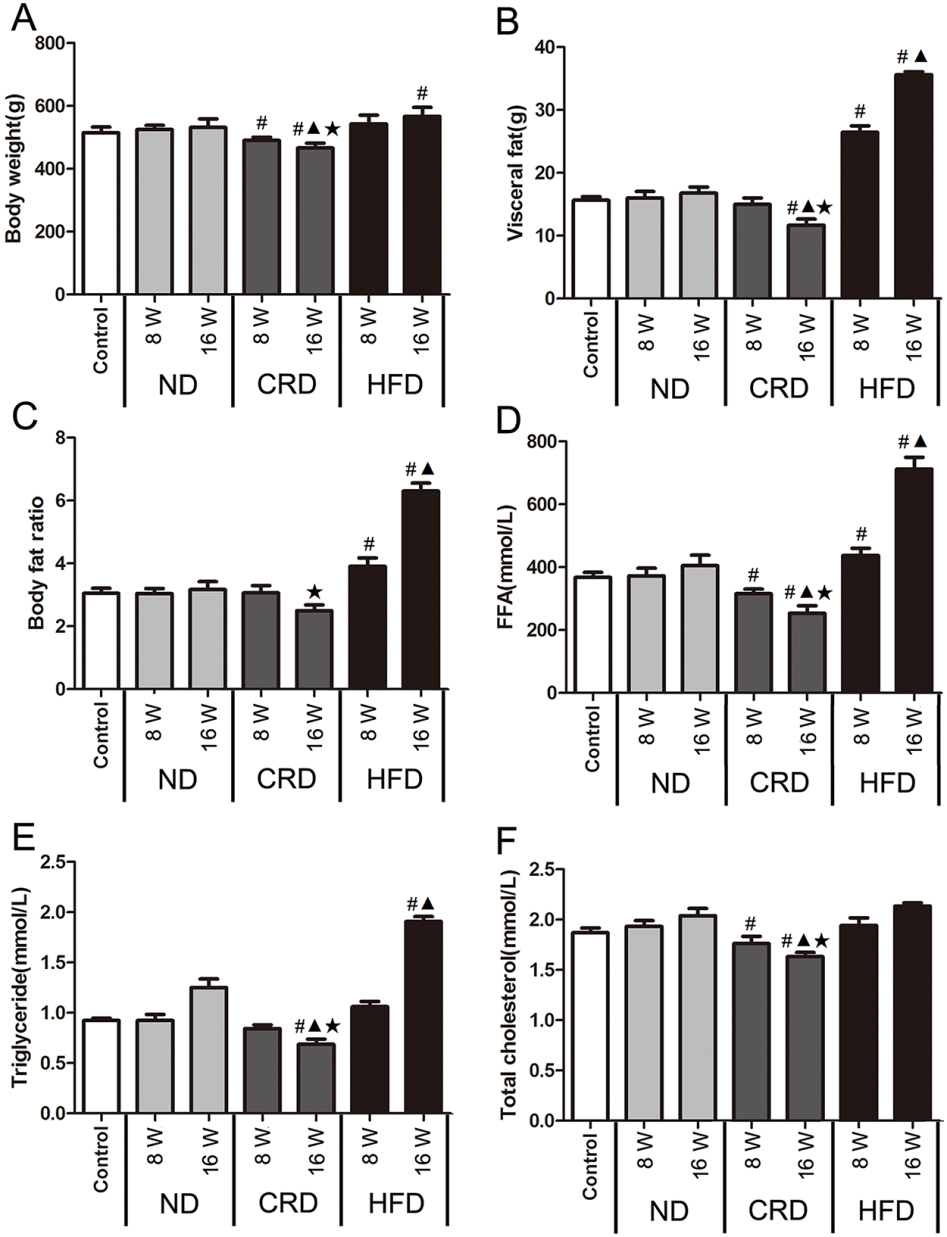
Changes in body composition and lipid levels in adult SD rats following dietary intervention (n = 5 for each group). The body weight (A), visceral fat (B), body fat ratio (C), plasma FFA levels (D), plasma triglyceride levels (E), and total plasma cholesterol (F) in the ND, HFD and CRD groups at 0, 8 and 16 weeks. #: vs. 0 week, **▲**: CRD/HFD vs. ND, ★ CRD vs. HFD. P<0.05. 0 weeks (14 months old), 8 weeks (16 months old), and 16 weeks (18 months old).

The changes in body weight (BW), visceral fat, body fat ratios, triglyceride levels, total cholesterol and FFAs are shown in Fig 4. Compared with the 14-month (0 week) samples, the BW of the adult rats decreased in the CRD group and increased in the HFD group (P<0.05) during the intervention period, and the extent of this change was more apparent in the CRD group than the HFD group (P<0.05) at 16 weeks. At 8 weeks, the visceral fat and body fat ratios were increased in the HFD group (P<0.05), whereas no decrease was observed in the CRD group. In the HFD group (compared with the week 0 results), the FFA levels were significantly increased after 16 weeks of intervention and were accompanied by a marked increase in triglyceride levels; however, no significant changes in total cholesterol levels were apparent. Finally, the administration of the CRD at 16 weeks significantly reduced the FFA, triglyceride and total cholesterol levels (P<0.05).

### Effects of CRD and HFD on FBG, plasma insulin, islet cell insulin, glucagon expression and β- and α-cell areas

As displayed in Table 2, the rats fed the CRD for 16 weeks displayed significantly reduced levels of FPG and Fins; therefore, the HOMA-IR (P< 0.05), ISI (P< 0.05), and HOMA-β (P< 0.05) levels were improved compared with those in the ND group. In contrast, after 16 weeks of intervention, the HFD group displayed slightly elevated levels of FPG (P>0.05 compared with the ND group), significantly higher levels of Fins and HOMA-IR (P< 0.05), and decreased ISI (P<0.05) without obvious changes in HOMA-β compared with those of the ND group.

**Table 2:** Effect of dietary intervention on FPG levels, serum insulin levels, ISI, HOMA-IR and HOMA-β in adult SD rats. Results represent the means ± S.D. (n = 5 for each group).

Group (age, month)	FPG (mmol/L)	Insulin (mmol/L)	ISI	HOMA-IR	HOMA-β
Control (14-)	5.43±0.21	20.58±1.26	0.009±0.001	4.90±0.67	211.40±19.44
ND (16-)	5.63±0.15	22.53±1.47	0.008±0.001	5.64±0.42	211.80±17.22
(18-)	5.87±0.25	23.84±0.77	0.008±0.001	6.22±0.37	202.95±22.24
CRD (16-)	5.13±0.21	17.65±0.75	0.011±0.001^#^	4.03±0.33	217.88±21.15
(18-)	4.87±0.15^▲^^★^	16.11±0.59^#^^▲^^★^	0.013±0.001^#^^▲^^★^	3.49±0.23^#^^▲^^★^	237.13±19.35^#^^▲^^★^
HFD (16-)	5.93±0.41	24.70±1.85^#^	0.007±0.001^#^	6.53±0.92^#^	205.53±24.39
(18-)	6.47±0.29^#^	29.28±1.06^#^^▲^	0.005±0.000^#^^▲^	8.42±0.68^#^^▲^	198.15±11.89

^**#**^: versus 0 week,

^▲^: CRD/HFD compared with ND,

^★^: CRD compared with HFD.

P< 0.05. 0 weeks (14 months old), 8 weeks (16 months old), 16 weeks (18 months old).

**Fig 5:**
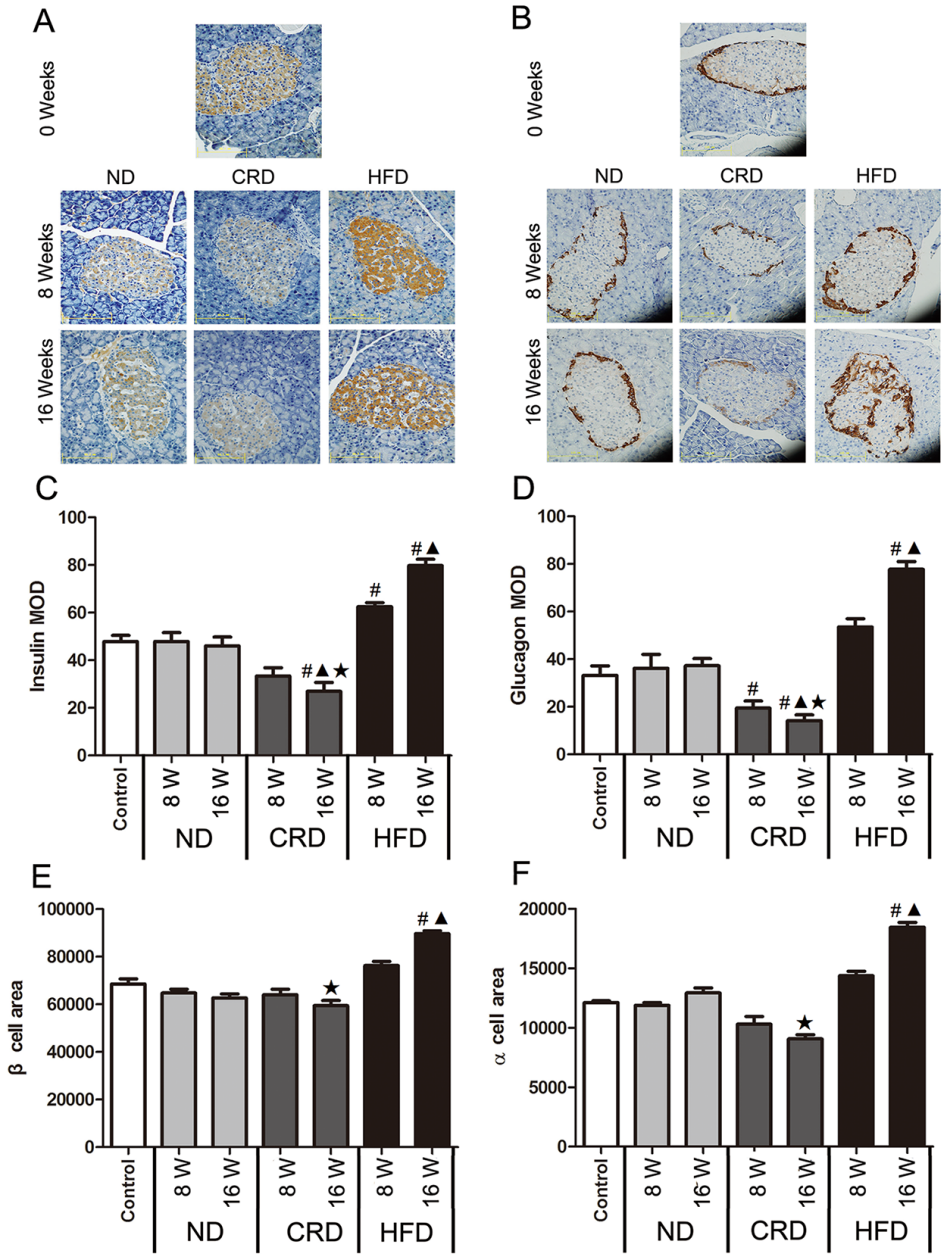
Effect of dietary intervention on the expression of insulin (A, brown staining) and glucagon (B, brown staining) in adult SD rat islet cells in the ND, HFD, and CRD groups at 0, 8, and 16 weeks, respectively (n = 5 for each group). Quantification of insulin (C) and glucagon (D) MOD and the area of the β (E) and α (F) cells. (400X, bars represent 125 μm). #: vs. 0 weeks, **▲**: CRD/HFD vs. ND, ★ CRD vs. HFD. P< 0.05. 0 weeks (14 months old), 8 weeks (16 months old), and 16 weeks (18 months old).

The insulin and glucagon levels in the islet cells are shown in Fig 5. Compared with the ND rats, the HFD-fed rats had a significant increase in insulin levels after 8 weeks of intervention (P< 0.05), and the glucagon levels and β- and α-cell areas were increased at 16 weeks (P< 0.05). However, the animals fed a CRD displayed a marked reduction in insulin levels and β- and α-cell areas at 16 weeks (P< 0.05). A significant reduction in glucagon was first observed at 8 weeks (P< 0.05).

## Discussion

We previously demonstrated that from 2 to 20–24 months of age, the appearance of autophagosomes and increased LC3B and LAMP2 expression (first observed at 14 months) in pancreatic islet cells coincided with a significant decrease in insulin secretion and elevated fasting blood glucose in aged rats without observable levels of apoptosis[14]. In this study, 14-month SD rats were chosen to receive a nutrient intervention strategy (HFD/CRD), which showed that after 16 weeks, the expression of autophagy-associated markers (LAMP2, LC3B) and AP increased in both the CRD- and HFD-fed rats. However, the apoptotic markers (TUNEL and c-caspase-3) were elevated only in the HFD-fed rats. We also observed that the expression of Beclin1 in islet cells from both the HFD and CRD intervention groups were increased. However, the expression of BCL2 and BCLXL increased only in the CRD group.

Xue[15] argued that both apoptosis and autophagy may be regulated by common factors because they share common signaling components, such as the Beclin1/BCL2 complex. Generally, autophagy blocks the induction of apoptosis, and apoptosis-associated caspase activation terminates the autophagic process[16, 17]. However, in special cases, autophagy or autophagy-relevant proteins may promote apoptosis[18], and autophagy has been shown to degrade the cytoplasm and lead to autophagic cell death[19–21]. One possibility is that BCL2 and BCLXL induce autophagy at low initial levels of stress (such as aging and moderate CRD) by activating the Beclin1-VPS34 complex when mitochondria are protected against lethal permeabilization. However, at higher, more advanced levels of stress (such as long-term HFD), BCL2 and BCLXL induce mitochondrial outer membrane permeabilization (MOMP) [22, 23], thereby initiating the apoptotic cascade[7]. The findings of the present study indicated that the expression of Beclin1 in islet cells increased in both the HFD and CRD intervention groups and that the expression of BCL2 and BCLXL increased in only the CRD group, which suggests the dissociation of the Beclin1/BCL2 complex. This dissociation may represent the inhibition of apoptosis under the stress of calorie-restricted intake, which also follows the low AI in the CRD group.

Compared with physiological aging, the CRD reduced body weight more rapidly (8 weeks) but did not improve insulin sensitivity (ISI, HOMA-IR and HOMA-β) during the same time; however, there was a tendency for lagging indicators (16 weeks). Although the HFD can increase insulin resistance in the short term (8 weeks), there were no significant BW changes during this time. The present study also showed that the earliest response to a HFD or CRD was a change in the FFA levels (at 8 weeks), which was evident before changes in triglycerides and total cholesterol were observed. Therefore, the level of FFA (due to different calorie intake) is a more intervention-sensitive energy indicator factor than are BW, triglycerides or total cholesterol in the transition from middle to later adulthood. Islet cells play a dual role in regulating glucose and an important role in aging[24, 25]. In the present study, we compared changes in the insulin and glucagon levels and α/β cell areas in response to a HFD and a CRD. Compared with physiological aging and a CRD, a HFD induced an increase in both plasma and β cell insulin levels in a relatively short period (8 weeks), and changes in α cell glucagon levels and the α cell area were apparent at 16 weeks. Therefore, the FFA, body fat ratio, and insulin/glucagon levels in islet cells may be associated with islet cell autophagy.

Basal and constitutive autophagy are important for the maintenance of normal islet function[26]. Autophagy and apoptosis control the turnover of organelles and proteins within cells and of cells within organisms, respectively, and numerous stress pathways within the islet cells sequentially elicit autophagy and apoptosis. In the present study (in the context of physiological aging), CRD-induced autophagy was markedly different from HFD-induced autophagy, as indicated by the expression of Beclin1, BCL2 and BCLXL. Moreover, the levels of islet cell insulin and glucagon, the α/β cell areas and the FFA levels were also markedly different. During insulin resistance, no correlation was previously identified between islet cell apoptosis/autophagy and the functional status of the pancreatic islet[27]. Our results revealed that the levels of islet cell insulin and glucagon, β/α cell areas and FFA levels may contribute to islet cell autophagy and/or apoptosis through the molecular regulation of Beclin1/BCL2. This result may partially explain why the islet cell dysfunction observed during physiological aging can be significantly improved by administering a CRD or further exacerbated by a HFD. Sustained, increased levels of autophagy and apoptosis of β or α cells combined with a HFD may lead to a loss of coordination between β and α cell masses with time. This requires further research.

We selected LC3B and LAMP2 as autophagy markers because the LC3B levels are thought to correlate with the level of mature autophagy membranes and are thus considered the most important markers during the expansion stage of autophagy[28, 29]. LAMP2 is required for the proper fusion of lysosomes with autophagosomes in the late stage of autophagy[30]. However, these markers are not α/β cell-specific. Additionally, LC3B and LAMP2 proteins are considered markers of autophagy maturation. Dynamic evidence, such as autophagic flux assessments, may be required to monitor the changes of autophagosomes. More signaling molecules warrant detection during this process.

In conclusion, our study demonstrated that from middle (14–16 months) to late adulthood (20 months), high levels of islet cell autophagy are associated with levels of FFA and insulin/glucagon expression within islets of rats. We believe that manipulating those factors could be useful for islet cell autophagy processes.

## Supporting Information

S1 Table Primary data of histogram in Fig 1C–1E. (DOCX)

S2 Table Primary data of histogram in Fig 2C and 2D. (DOCX)

S3 Table Primary data of histogram in Fig 3D–3F. (DOCX)

S4 Table Primary data of histogram in Fig 4A–4F. (DOCX)

S5 Table Primary data of histogram in Fig 5C–5F. (DOCX)
