# Supplementary material for: Factors that Affect Pancreatic Islet Cell Autophagy in Adult Rats: Evaluation of a Calorie-Restricted Diet and a High-Fat Diet
Source: PLoS One. 2016 Mar 10;11(3):e0151104. doi: 10.1371/journal.pone.0151104 (PMC4786268; doi:10.1371/journal.pone.0151104)
Supplement: S1 Table — (DOCX) [file pone.0151104.s001.docx]

**S1 Table. Primary data of histogram in Figure 1C-1E.** Effect of dietary intervention on islet cell AP activity (C), LC3B (D) and LAMP2 (E) expression in adult SD rats. Results represent the means ± S.D. (n=5 for each group).

| Group | AP activity | LC3B/GAPDH | LAMP2/GAPDH |
| --- | --- | --- | --- |
| (age, month) |  |  |  |
| ND (14-) | 1.00±0.03 | 0.45±0.03 | 0.41±0.01 |
| (16-) | 1.06±0.04 | 0.47±0.02 | 0.42±0.02 |
| (18-) | 1.14±0.06 | 0.51±0.03 | 0.45±0.02 |
| CRD (14-) | 1.00±0.04 | 0.43±0.02 | 0.43±0.01 |
| (16-) | 1.17±0.06 | 0.57±0.04 | 0.45±0.03 |
| (18-) | 1.38±0.05^＃▲^ | 0.65±0.04^＃▲^ | 0.60±0.02^＃▲^ |
| HFD (14-) | 1.00±0.02 | 0.47±0.02 | 0.39±0.01 |
| (16-) | 1.27±0.05 | 0.60±0.02 | 0.57±0.02^＃^ |
| (18-) | 1.49±0.06^＃▲^ | 0.80±0.02^＃▲^ | 0.71±0.02^＃▲^ |

**＃: versus 0 week, ▲: CRD/HFD compared with ND, ★**:**CRD compared with HFD. P< 0.05. 0 weeks (14 months old), 8 weeks (16 months old), 16 weeks (18 months old).**
