# Supplementary material for: Factors that Affect Pancreatic Islet Cell Autophagy in Adult Rats: Evaluation of a Calorie-Restricted Diet and a High-Fat Diet
Source: PLoS One. 2016 Mar 10;11(3):e0151104. doi: 10.1371/journal.pone.0151104 (PMC4786268; doi:10.1371/journal.pone.0151104)
Supplement: S2 Table — (DOCX) [file pone.0151104.s002.docx]

**S2 Table. Primary data of histogram in Figure 2 C and 2D.** Effects of dietary intervention on islet cell AI (C) and c-caspase-3 (D) in adult SD rats. Results represent the means ± S.D. (n=5 for each group).

| Group | AI | c-caspase-3 MOD |
| --- | --- | --- |
| (age, month) |  |  |
| ND (14-) | 3.22±0.59 | 481.58±18.53 |
| (16-) | 3.47±0.57 | 496.42±22.20 |
| (18-) | 3.61±0.49 | 533.71±28.00 |
| CRD (14-) | 3.27±0.55 | 485.65±19.02 |
| (16-) | 2.73±0.34 | 413.78±22.24 |
| (18-) | 1.88±0.35^＃▲★^ | 289.68±31.76^＃▲★^ |
| HFD (14-) | 3.18±0.63 | 478.23±17.88 |
| (16-) | 4.35±0.59 | 641.72±26.41^#^ |
| (18-) | 7.50±0.99^＃▲^ | 757.67±21.48^＃▲^ |

**＃: versus 0 week, ▲: CRD/HFD compared with ND, ★: CRD compared with HFD. P< 0.05. 0 weeks (14 months old), 8 weeks (16 months old), 16 weeks (18 months old).**
