# Supplementary material for: Factors that Affect Pancreatic Islet Cell Autophagy in Adult Rats: Evaluation of a Calorie-Restricted Diet and a High-Fat Diet
Source: PLoS One. 2016 Mar 10;11(3):e0151104. doi: 10.1371/journal.pone.0151104 (PMC4786268; doi:10.1371/journal.pone.0151104)
Supplement: S3 Table — (DOCX) [file pone.0151104.s003.docx]

**S3 Table. Primary data of histogram in Figure 3D- 3F**. Effect of dietary intervention on the expressions of Beclin1 (D), BCL2 (E), and BCLXL (F) in the islet cells of adult SD rats. Results represent the means ± S.D. (n=5 for each group).

| Group | Beclin1/GAPDH | BCL2/GAPDH | BCLXL/GAPDH |
| --- | --- | --- | --- |
| (age, month) |  |  |  |
| ND (14-) | 0.31±0.01 | 0.64±0.02 | 0.35±0.01 |
| (16-) | 0.32±0.02 | 0.61±0.03 | 0.32±0.01 |
| (18-) | 0.33±0.01 | 0.56±0.02 | 0.29±0.02 |
| CRD (14-) | 0.29±0.01 | 0.60±0.01 | 0.33±0.02 |
| (16-) | 0.35±0.01 | 0.76±0.04 | 0.43±0.02 |
| (18-) | 0.41±0.01^▲^ | 0.89±0.03^＃▲★^ | 0.51±0.02^＃▲★^ |
| HFD (14-) | 0.33±0.02 | 0.68±0.03 | 0.37±0.01 |
| (16-) | 0.38±0.01 | 0.52±0.03 | 0.28±0.02 |
| (18-) | 0.44±0.01^＃▲^ | 0.38±0.02^＃▲^ | 0.20±0.01^＃▲^ |

**＃: versus 0 week, ▲: CRD/HFD compared with ND, ★ CRD compared with HFD. P< 0.05. 0 weeks (14 months old), 8 weeks (16 months old), 16 weeks (18 months old).**
