# Supplementary material for: Factors that Affect Pancreatic Islet Cell Autophagy in Adult Rats: Evaluation of a Calorie-Restricted Diet and a High-Fat Diet
Source: PLoS One. 2016 Mar 10;11(3):e0151104. doi: 10.1371/journal.pone.0151104 (PMC4786268; doi:10.1371/journal.pone.0151104)
Supplement: S4 Table — (DOCX) [file pone.0151104.s004.docx]

**S4 Table. Primary data of histogram in Figure 4A- 4F**. Changes in body composition (A,B,C) and lipid levels (D,E,F) in adult SD rats following dietary intervention. Results represent the means ± S.D. (n=5 for each group).

| Group | Body Weight | Visceral Fat | Body fat ratio(%) | FFA | TG | TC |
| --- | --- | --- | --- | --- | --- | --- |
| (age, month) |  |  |  |  |  |  |
| ND (14-) | 515.00±18.03 | 15.74±1.69 | 3.04±0.28 | 367.33±39.55 | 0.92±0.12 | 1.87±0.08 |
| (16-) | 525.00±13.23 | 15.99±1.83 | 3.04±0.28 | 372.33±42.85 | 0.92±0.11 | 1.93±0.10 |
| (18-) | 531.67±27.54 | 16.79±1.68 | 3.17±0.43 | 405.00±57.24 | 1.25±0.15 | 2.04±0.13 |
| CRD (14-) | 522.00±19.01 | 15.88±1.71 | 3.02±0.25 | 372.45±41.03 | 0.91±0.10 | 1.88±0.09 |
| (16-) | 490.00±10.00^#^ | 14.98±1.78 | 3.06±0.40 | 315.67±25.03^#^ | 0.84±0.07 | 1.76±0.13^#^ |
| (18-) | 466.67±15.28^＃▲★^ | 11.67±1.68^＃▲★^ | 2.50±0.33^★^ | 253.67±41.79^＃▲★^ | 0.69±0.09^＃▲★^ | 1.63±0.07^＃▲★^ |
| HFD (14-) | 508.33±16.89 | 15.61±1.64 | 3.06±0.32 | 362.58±38.25 | 0.93±0.13 | 1.85±0.07 |
| (16-) | 542.67±28.22 | 26.44±1.82^＃^ | 3.91±0.46^＃^ | 437.33±39.27^＃^ | 1.06±0.09 | 1.94±0.13 |
| (18-) | 566.67±28.87^＃^ | 35.62±0.80^＃▲^ | 6.30±0.44^＃▲^ | 712.33±64.78^＃▲^ | 1.91±0.09^＃▲^ | 2.14±0.05 |

**＃: versus 0 week, ▲: CRD/HFD compared with ND, ★: CRD compared with HFD. P< 0.05. 0 weeks (14 months old), 8 weeks (16 months old), 16 weeks (18 months old).**
