# Supplementary material for: Factors that Affect Pancreatic Islet Cell Autophagy in Adult Rats: Evaluation of a Calorie-Restricted Diet and a High-Fat Diet
Source: PLoS One. 2016 Mar 10;11(3):e0151104. doi: 10.1371/journal.pone.0151104 (PMC4786268; doi:10.1371/journal.pone.0151104)
Supplement: S5 Table — (DOCX) [file pone.0151104.s005.docx]

**S5 Table. Primary data of histogram in Figure 5C- 5F.** Effect of dietary intervention on the expression of insulin (C),glucagon (D),the area of the β (E) and α (F) cells in adult SD rat islet cells in the ND, HFD, and CRD groups at 0, 8, and 16 weeks, respectively. Results represent the means ± S.D. (n=5 for each group).

| Group | Insulin MOD | Glucagon MOD | β cell area | α cell area |
| --- | --- | --- | --- | --- |
| (age, month) |  |  |  |  |
| ND (14-) | 47.90±4.47 | 33.23±6.82 | 68543.33±3735.01 | 12102.33±259.58 |
| (16-) | 47.86±6.50 | 36.21±10.09 | 64763.00±2788.14 | 11875.67±409.20 |
| (18-) | 46.07±6.40 | 37.30±5.12 | 62740.00±2822.76 | 12923.00±724.58 |
| CRD (14-) | 48.32±4.53 | 32.78±6.51 | 68275.45±3684.21 | 12698.84±275.97 |
| (16-) | 33.37±6.11 | 19.42±5.32^#^ | 64061.67±4005.7 | 10299.00±1113.21 |
| (18-) | 26.99±6.50^＃▲★^ | 14.19±4.20^＃▲★^ | 59475.33±3694.64^★^ | 9095.33±631.12^★^ |
| HFD (14-) | 47.23±4.40 | 33.82±7.03 | 68759.33±3805.42 | 11695.67±238.41 |
| (16-) | 62.57±2.87^＃^ | 53.51±6.16 | 76304.00±3008.96 | 14356.00±685.57 |
| (18-) | 79.83±4.49^＃▲^ | 77.78±5.63^＃▲^ | 89725.00±1998.67^＃▲^ | 18448.67±671.28^＃▲^ |

**＃: versus 0 week, ▲: CRD/HFD compared with ND, ★: CRD compared with HFD. P< 0.05. 0 weeks (14 months old), 8 weeks (16 months old), 16 weeks (18 months old).**
